# Effects of small-dose remifentanil combined with index of consciousness monitoring on gastroscopic polypectomy: a prospective, randomized, single-blinded trial

**DOI:** 10.1186/s13063-018-2783-4

**Published:** 2018-07-18

**Authors:** Minqiang Liu, Hongyan Wu, Danling Yang, Fengxian Li, Zhichao Li, Song Wang, Renliang He

**Affiliations:** 1 Department of Anesthesiology, Shenzhen Third People’s Hospital, No. 29 Bulan Road, Longgang District, Shenzhen, 518112 Guangdong China; 2 Department of Endoscopy, Shenzhen Third People’s Hospital, No. 29 Bulan Road, Longgang District, Shenzhen, 518112 Guangdong China; 3 Department of Anesthesiology, Zhujiang Hospital of Southern Medical University, No. 253 Middle Industrial Avenue, Haizhu District, Guangzhou, 518112 Guangdong China

**Keywords:** Remifentanil, Index of consciousness, Gastroscopy, Polypectomy

## Abstract

**Background:**

With the development of painless diagnosis and treatment, remifentanil, a synthetic opioid agonist, is increasingly used in gastroscopy for its rapid, short-term, and potent analgesic effect. However, the dosage of remifentanil used in endoscopy is unclear. Index of consciousness (IOC) is a new anesthesia depth-monitoring indicator that can be divided into index of consciousness 1 (IOC_1_) and index of consciousness 2 (IOC_2_); IOC_1_ is used for estimating a patient’s sedation state, whereas IOC_2_ reflects analgesic depth. We hypothesized that combining with IOC_1_ and IOC_2_ monitoring may be helpful to identify an optimal remifentanil dosage in gastroscopic polypectomy.

**Methods:**

One hundred twenty patients scheduled for gastroscopic polypectomy were enrolled and were randomly assigned to remifentanil 2 ng/mL (group R2), 4 ng/mL (group R4), or 6 ng/mL (group R6), and 40 cases were in each group. During the anesthesia period, remifentanil was kept at the initial given concentration but propofol was adjusted according to IOC_1_. The primary outcomes were the dosage of propofol and remifentanil. The secondary outcomes were the variety of IOC_1_ and IOC_2_, patients’ awakening time, and peri-operative adverse reactions such as hypotension, hypertension, bradycardia, tachycardia, body movements, hypoxemia, therapy interruption, nausea, vomiting, aspiration, and intra-operative awareness.

**Results:**

With the increasing dosage of remifentanil, the propofol dosage and patients’ awakening time decreased significantly, the morbidity of hypertension and body movements also declined, but the incidence of hypotension, bradycardia, and hypoxemia rose. In group R2, the value of IOC_2_ remained above 50 during the treatment. However, IOC_2_ dropped to below 30 at the beginning of the gastroscopy in group R6, and there was statistical difference in hypoxemia between groups R2 and R6 (*P* <0.05).

**Conclusions:**

With the help of IOC monitoring, we found that a target concentration of remifentanil 4 ng/mL is comparatively ideal in patients under gastroscopic polypectomy.

**Trial registration:**

Chinese Clinical Trial Register: ChiCTR-OOD-16009489, on October 19, 2016.

## Background

Gastric polyp is the bulging of the gastric mucosa, usually occurring in the gastric antrum [1]. The patient may have upper abdominal pain, abdominal distension, dysphagia, or loss of appetite or may have no discomfort; nowadays, the major diagnostic method is gastroscopy [2]. Because the most common histologic type of gastric polyp is the hyperplastic polyp, which has certain risks of turning into a malignant tumor [3], the main therapy for this disease is gastroscopic polypectomy [1, 4]. However, endoscopic therapy is usually associated with certain adverse reactions such as nervousness, nausea, vomiting, and choking cough [5, 6], and severe discomfort such as coughing or body movements may lead to aggravation of a pre-existing condition or even interruption of treatment, especially in some critical patients with physiological dysfunction (for instance, respiratory disease or cardiovascular disease) [7]. Therefore, finding a suitable way to enhance patient compliance is very important.

It has been reported that the administration of intravenous anesthesia can effectively inhibit upper airway reflex, eliminate patients’ anxiety, and improve patients’ comfort during endoscopy, which led to an increase of patients’ willingness to undergo follow-up gastroscopic examination or treatment [8, 9]. In previous studies, a number of reports have confirmed the efficacy of general anesthesia in gastrointestinal endoscopy [10–12], and remifentanil proved to be a safe and effective opioid receptor agonist during endoscopy because of its effective inhibition of autonomic nervous reflex in the upper respiratory tract and rare adverse effects on the cardiovascular and respiratory systems [13–15]. However, few reports explore the optimal dose of remifentanil during gastroscopic treatment. With the development of anesthesiology, intra-operative anesthesia depth monitoring is increasingly being used in the clinic. Index of consciousness (IOC) is a new indicator that can effectively measure patients’ sedation depth and analgesic state [16]. IOC can be divided into index of consciousness 1 (IOC_1_) and index of consciousness 2 (IOC_2_); IOC_1_ is used for estimating a patient’s sedation state, whereas IOC_2_ reflects analgesic depth [17]. Previous studies have shown that accurate anesthesia depth monitoring can help optimize the use of anesthetics, shorten the recovery time, and reduce post-operative analgesia complications [18, 19]. In this research, we combined both IOC_1_ and IOC_2_ monitoring, as the validity of IOC_1_ in the evaluation of sedation depth has been confirmed, we hypothesized that IOC_2_monitoring may help in determining an appropriate dosage of remifentanil in gastroscopic polypectomy.

## Methods

### Study design and setting

**Fig. 1:**
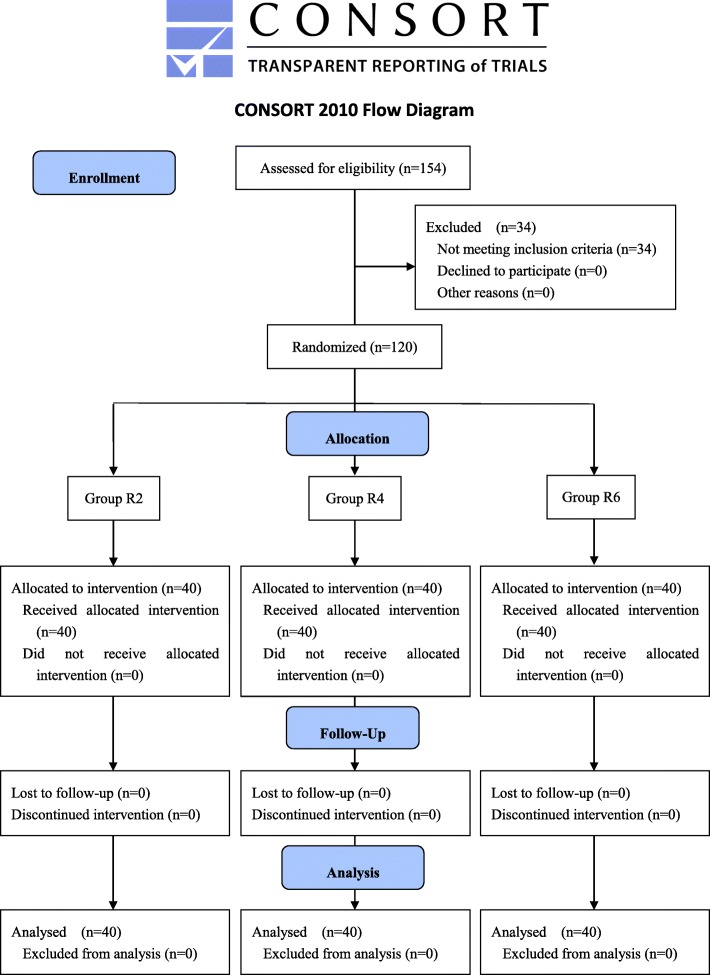
Flow diagram of the structure of the study

This study was a prospective, randomized, single-blind trial. It was conducted with the approval of the medical ethics committee of Shenzhen Third People’s Hospital (No. 29 Bulan Road, Longgang District, Shenzhen, Guangdong, China, approval number 2016–001; October 17, 2016) and was registered in the Chinese Clinical Trial Register (http://www.chictr.org.cn/index.aspx, registration number ChiCTR-OOD-16009489; October 19, 2016). Patients of American Society of Anesthesiologists (ASA) class I or II, undergoing selective gastroscopic polypectomy, were recruited. Written informed consent was obtained and documented for all participants before anesthesia. The exclusion criteria were those older than 60 years or younger than 18 years, pregnancy, body weight exceeding 20% of the ideal body weight, active upper gastrointestinal bleeding, a history of bronchial asthma or chronic obstructive pulmonary disease, upper airway infection in the previous 2 weeks, impaired kidney or liver functions, drug abuse, a known hypersensitivity to propofol or remifentanil, and patients who were expected to have difficult airway intubation or an expected operation time exceeding 30 min. Furthermore, participants would be excluded after recruitment if the anesthesia protocol or endoscopic therapy was temporarily changed for any reason. The structure of the study is illustrated in Fig. 1.

### Anesthesia

All patients fasted for 10 h but received 10 mL of oral dyclonine (dyclonine hydrochloride mucilage, batch number 17011021, Yangtze River Pharmaceutical Co., Ltd., Handan, China) 15 min prior to entering the gastroscopy room. After the patient’s admission, IOC_1_ and IOC_2_ were monitored continuously. Electrocardiogram (ECG), non-invasive blood pressure (NIBP), and pulse oxygen saturation (SpO_2_) were constantly monitored by using the GE Datex-Ohmeda S/5™ anesthesia work station (ADU, Datex-Ohmeda Inc., Madison, WI, USA). Peripheral venous access was secured by using a 24-G intravenous needle on the right dorsal hand, a left-side position was taken, and normal saline was infused at a rate of 10 mL·kg^− 1^·h^− 1^. All anesthetics were administered via a target-controlled infusion system (Fresenius Kabi Base Primea, Fresenius Kabi Pharmaceutical Co. Ltd., Bad Homburg, Germany). Propofol (Propofol Medium and Long Chain Fat Emulsion Injection, batch number 16Kl5866, Fresenius Kabi Austria GmbH, Graz, Austria) was administered at an effect-site concentration of about 2 to 6 μg/mL (Marsh model) 10 min after the patient’s admission. When IOC_1_ was stably maintained at about 40 to 60 for at least 1 min, remifentanil (remifentanil hydrochloride for injection, batch number 6161009, Yichang Humanwell Pharmaceutical, Hubei, China) was infused with a plasma concentration of 2, 4, and 6 ng/mL (Minto model) in groups R2, R4, and R6, respectively. Gastroscopy was carried out after remifentanil reached the target concentration for 2 min. During the whole procedure, remifentanil was kept at the initial given concentration; the anesthesia depth was mainly controlled by propofol, which was adjusted on the basis of the variety of IOC_1_ values. When IOC_1_ was maintained between 40 and 60, the current propofol concentration was maintained. If the IOC_1_ values exceeded 60, the propofol dose was increased by 0.5 μg/mL per adjustment, whereas if IOC_1_ went below 40, the propofol concentration was decreased by 0.5 μg/mL per adjustment.

### IOC monitoring

We used a multi-parameter anesthesia monitor (Angel-60,000, Shenzhen Weihaokang Medical Technology Co., Ltd., Guangdong, China) for IOC monitoring. After skin cleaning with normal saline, the red, yellow, and green electrodes were connected to the center of the forehead, the top right of the brow ridge, and the cheekbone on the same side, respectively. IOC_1_ and IOC_2_ were recorded automatically every 15 s.

### Complications and management

During the peri-operative period, hypotension was defined as a decrease in mean arterial pressure (MAP) over 20% or NIBP of not more than 90/60 mm Hg [20, 21]; in this case, a dose of about 0.5 to 1 mg of metaraminol (metaraminol bitartrate injection, batch number 17020438, Beijing Yokon Pharmaceutical Co., Ltd., Beijing, China) was injected. Hypertension was defined as MAP increased over 20% or NIBP of at least 140/90 mm Hg [22, 23]; in this case, an injection of about 10 to 20 mg of urapidil (urapidil injection, batch number 16112201, Guangzhou ImVin Pharmaceutical Co., Ltd., Guangzhou, China) was given. When heart rate (HR) went below 50 beats/min, bradycardia was diagnosed [24], and an injection of about 0.5 to 1 mg of atropine (atropine sulfate injection, batch number 1701091, Tianjin Kingyork Pharmaceutical Co., Ltd., Tianjin, China) was given. When HR stayed above 100 beats/min, tachycardia was diagnosed [25], and a dose of about 0.5 to 1 mg/kg of esmolol (esmolol hydrochloride injection, batch number 6F0142C05, Qilu Pharmaceutical Co., Ltd., Jinan, China) was injected. Moreover, if SpO_2_ dropped below 95%, the temporomandibular joint should be lifted; if SpO_2_ dropped below 90%, hypoxemia was diagnosed [26, 27]; and if hypoxemia lasted over 2 min, all drugs and operations must stop, assisted breathing with a balloon mask must be provided, and the trial must be terminated. In addition, the interval between pulling out the gastroscope and opening the eyes when calling the patient’s name in a normal voice was defined as the awakening time [28, 29]. Whether the patient could partially or totally remember the events during the whole general anesthesia period was defined as intra-operative awareness [30, 31].

### Sample size calculation

The calculation of the sample size in this research was based on the literature reports related to remifentanil used in gastroscopy [13–15, 32, 33]. The average cases were about 32 to 42 patients in each group. Thus, we decided to enroll 40 cases in each group. Considering the possible loss of cases in the study (about 20% to 30%), we recruited a total of 154 patients (Fig. 1).

### Randomization and blinding

The group allocation was based on a random number generated by a computer and divided by 3; if the remainder was 1, the patient was allocated to the remifentanil 2 ng/mL group (group R2); if the remainder was 2, the patient was assigned to the remifentanil 4 ng/mL group (group R4); otherwise, the patient was assigned to the remifentanil 6 ng/mL group (group R6). The anesthesiologists involved in the practice of anesthesia knew the patient’s group allocation, but all parameters were observed and recorded by another anesthesiologist who was blind to the group allocation and did not take part in the implementation of anesthesia.

### Data collection

The average target concentration of propofol and patient’s awakening time were observed. The values of IOC_1_, IOC_2_, MAP, HR, and SpO_2_ were monitored during the entire course of therapy and were recorded at the following time points: 10 min after patients entered the endoscopy room (T_0_), at the beginning of the gastroscopy (T_1_), at the time of the polypectomy (T_2_), at the end of the operation (T_3_), and at the time the patient opened their eyes after being called by name (T_4_). Peri-operative adverse reactions such as hypotension, hypertension, bradycardia, tachycardia, body movements, hypoxemia, interruption of therapy, nausea, vomiting, aspiration, and intra-operative awareness were also observed.

### Outcomes

The primary outcomes were the dosage of propofol and remifentanil. The secondary outcomes were the variety of IOC_1_ and IOC_2_, patients’ awakening time, and peri-operative adverse reactions such as hypotension, hypertension, bradycardia, tachycardia, body movements, hypoxemia, therapy interruption, nausea, vomiting, aspiration, and intra-operative awareness. Other outcomes were MAP, HR, and SpO_2_ at different time points.

### Statistical analysis

All data were analyzed by using the SPSS statistical package (SPSS 13.0 for Windows, Inc., Chicago, IL, USA) and were presented as mean ± standard deviation, number, or percentage. Group comparisons about age, weight, height, operation time, propofol dosage, and awakening time were analyzed by using one-way analysis of variance (ANOVA). Values of IOC_1_, IOC_2_, MAP, HR, and SpO_2_ were measured by repeated-measures ANOVA. Categorical data were compared by using the chi-squared test or Fisher’s exact test. A *P* value of less than 0.05 was considered statistically significant.

## Results

One hundred fifty-four patients scheduled for gastroscopic polypectomy were initially assessed for eligibility from February 22, 2017 to June 30, 2017. Thirty-four patients were excluded: 26 patients were excluded for being older than 60 years, three because of body weight exceeding 20% of the ideal body weight, three had tachycardia, one had bradycardia, and one had had upper airway infection in the previous 2 weeks. No severe adverse event leading to a termination of the study was observed. Therefore, altogether 120 patients (40 in each group) were enrolled in this study (Fig. 1).

### Demographic profile

Elements of the patients’ demographic profile, including sex, age, weight, height, and operation time, were similar among groups (*P* >0.05) (Table 1).

**Table 1:** Demographics of the three groups

Item	Group R2	Group R4	Group R6	*P* value
Sex, male/female	22/18	24/16	23/17	0.903
Age, years	45 ± 10	43 ± 10	45 ± 8	0.442
Height, cm	164 ± 9	165 ± 8	164 ± 8	0.892
Weight, kg	62 ± 10	61 ± 11	60 ± 9	0.668
Operation time, min	18 ± 5	18 ± 4	18 ± 4	0.782

### Propofol dosage and awakening time

As shown in Table 2, the average propofol dosage decreased significantly with the increasing dose of remifentanil (*P* <0.01), and the awakening time in groups R4 and R6 also decreased when compared with group R2 (*P* <0.05). However, there were no statistical differences in propofol dose and awakening time between groups R4 and R6 (*P* >0.05).

**Table 2:** Comparison of propofol dosage and awakening time among groups

Item	Group R2	Group R4	Group R6	*P* value
Average propofol dosage, μg/mL	2.6 ± 0.6	2.2 ± 0.6^**^	2.1 ± 0.5^**^	0.001
Awakening time, min	3.1 ± 1.1	2.4 ± 1.2^*^	2.5 ± 1.2^*^	0.025

Compared with group R2, ^*^P <0.05, ^**^P <0.01

### IOC_1_ in the three groups

**Fig. 2:**
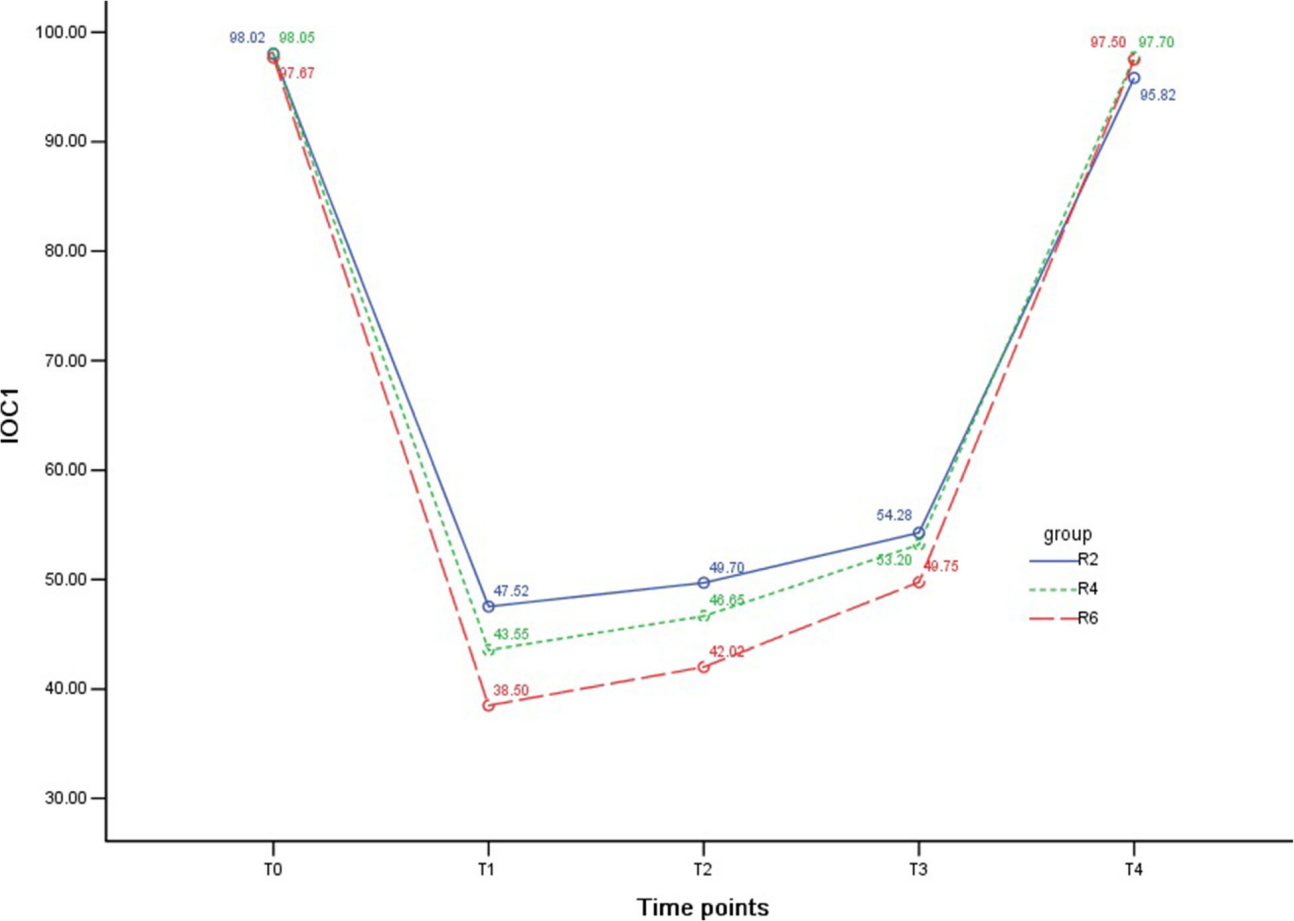
Index of consciousness 1 (IOC_1_) in the three groups. The variety of IOC_1_ at different time points in the three groups is shown

As shown in Table 3 and Fig. 2, IOC_1_ decreased significantly after anesthesia induction in all groups (*P* <0.01) and then gradually increased to the baseline after the operation but was maintained at a comparatively lower state at T_4_ in group R2 (*P* <0.05). Compared with group R2, IOC_1_ was lower at T_1_ and T_4_ in group R4 (both *P* <0.05) and relatively lower at T_1_, T_2_, T_3_, and T_4_ in group R6 (all *P* <0.05). IOC_1_ was also lower at T_1_ and T_2_ in group R6 compared with group R4 (both *P* <0.01).

**Table 3:** Comparison of index of consciousness among groups

Item	Time point	Group R2	Group R4	Group R6	*P* value
IOC_1_	T_0_	98.0 ± 1.5	98.1 ± 1.3	97.7 ± 1.3	0.371
	T_1_	47.5 ± 6.9^&&^	43.6 ± 9.5^&&*^	38.5 ± 7.27^&&**##^	0.000
	T_2_	49.7 ± 7.1^&&^	46.7 ± 8.8^&&^	42.0 ± 6.1^&&**##^	0.000
	T_3_	54.3 ± 9.0^&&^	53.2 ± 8.3^&&^	49.8 ± 7.9^&&*^	0.046
	T_4_	95.8 ± 4.5^&^	97.7 ± 1.4^**^	97.5 ± 1.5^**^	0.006
IOC_2_	T_0_	97.8 ± 1.9^&&^	97.8 ± 1.5	98.0 ± 1.2	0.753
	T_1_	47.2 ± 7.9^&&^	32.9 ± 9.5^&&**^	26.6 ± 7.4^&&**##^	0.000
	T_2_	53.0 ± 11.5^&&^	37.1 ± 11.5^&&**^	34.2 ± 8.5^&&**^	0.000
	T_3_	59.6 ± 16.1^&&^	46.0 ± 14.3^&&**^	45.6 ± 11.5^&&**^	0.000
	T_4_	98.2 ± 1.1	98.0 ± 0.9	97.8 ± 1.4	0.274

Compared with group T_0_, ^&^*P* <0.05, ^&&^*P* <0.01; compared with group R2, ^*^P <0.05, ^**^P <0.01; compared with group R4, ^#^*P* <0.05, ^##^*P* <0.01

Abbreviations: *IOC*_*1*_ index of consciousness 1, *IOC*_*2*_ index of consciousness 2

### IOC_2_ in the three groups

**Fig. 3:**
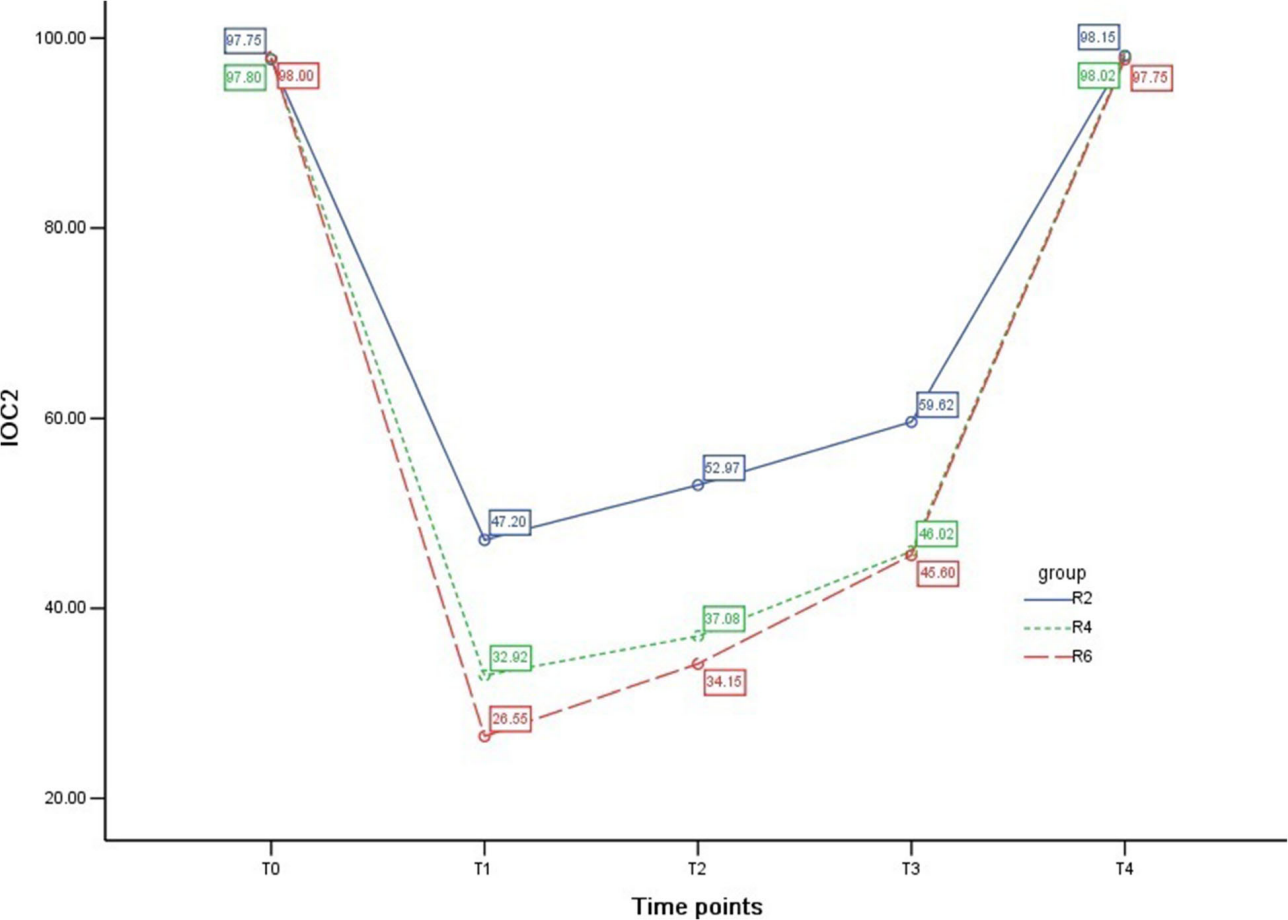
Index of consciousness 2 (IOC_2_) in the three groups. The variety of IOC_2_ at different time points in the three groups is shown

As shown in Table 3 and Fig. 3, IOC_2_ dropped significantly after general anesthesia induction in the three groups (*P* <0.01) and then came back to the basic level at T_4_. In group R2, IOC_2_ remained above 50 at T_1_ and T_2_. In group R6, IOC_2_ went below 30 at T_1_. Compared with group R2, IOC_2_ was remarkably lower at T_1_, T_2_, and T_3_ than that in groups R4 and R6 (all *P* <0.01). IOC_2_ was also relatively lower at T_1_ than that in group R6 compared with group R4 (*P* <0.01).

### Peri-operative adverse reactions among groups

As shown in Table 4, with the increasing dosage of remifentanil, the morbidity of hypertension and body movements declined, but the incidence of hypotension, bradycardia, and hypoxemia rose, and there were statistical differences in hypertension and hypoxemia between groups R2 and R6 (*P* <0.05). However, no statistically significant differences in hypotension, bradycardia, tachycardia, and body movements were observed among the three groups (*P* >0.05). Furthermore, no patient in any group had therapy interruption, nausea, vomiting, aspiration, or intra-operative awareness.

**Table 4:** Peri-operative adverse reactions

Item	Group R2	Group R4	Group R6	*P* value
Hypotension (%)	7 (17.5%)	8 (20.0%)	12 (30.0%)	0.367
Hypertension (%)	6 (15.0%)	4 (10.0%)	0 (0) ^*^	0.047
Bradycardia (%)	5 (12.5%)	7 (17.5%)	8 (20.0%)	0.657
Tachycardia (%)	6 (15.0%)	5 (12.5%)	6 (15.0%)	0.934
Body movement(%)	2 (5.0%)	1 (2.5%)	0 (0)	0.359
Hypoxemia (%)	1 (2.5%)	4 (10.0%)	8 (20.0%)^*^	0.041
Therapy interruption (%)	0 (0)	0 (0)	0 (0)	1.000
Nausea (%)	0 (0)	0 (0)	0 (0)	1.000
Vomiting (%)	0 (0)	0 (0)	0 (0)	1.000
Aspiration (%)	0 (0)	0 (0)	0 (0)	1.000
Intra-operative awareness (%)	0 (0)	0 (0)	0 (0)	1.000

Compared with group R2, ^*^P <0.05

### MAP among groups

**Fig. 4:**
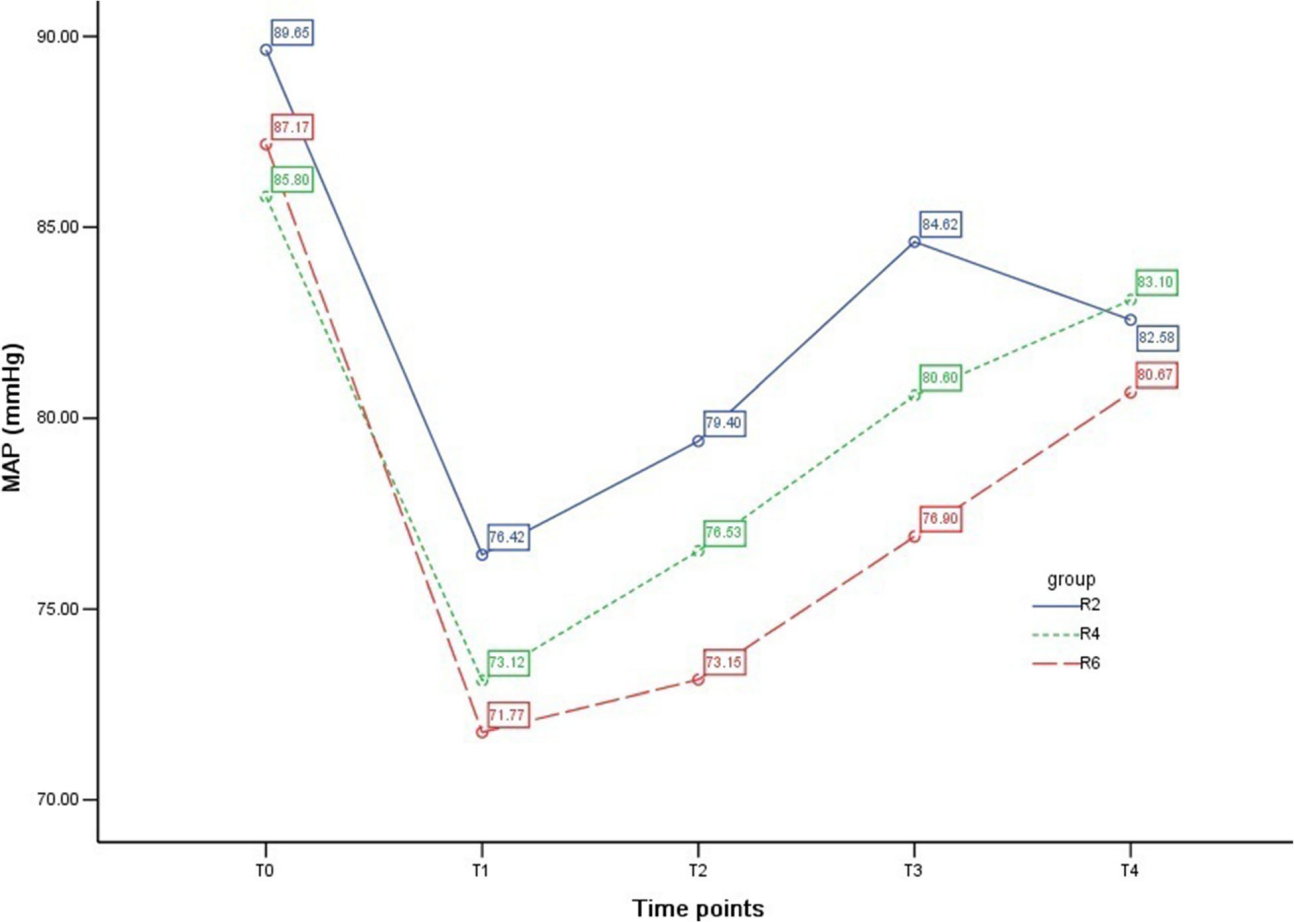
Mean arterial pressure (MAP) among groups. The variety of MAP at different time points in the three groups is shown

Compared with T_0_, MAP decreased after anesthesia induction in all groups (*P* <0.01) and gradually increased as the operation progressed but was still lower than the baseline value at T_4_ in groups R2 and R6 (*P* <0.01). Compared with group R2, MAP was comparatively lower at T_1_ in group R4 (*P* <0.05) and relatively lower at T_1_, T_2_, and T_3_ in group R6 (all *P* <0.01) (Table 5 and Fig. 4).

**Table 5:** Comparison of MAP, HR, and SpO_2_ in the three groups

Item	Time point	Group R2	Group R4	Group R6	*P*
MAP, mm Hg	T_0_	89.7 ± 8.7	85.8 ± 9.5	87.2 ± 9.1	0.162
	T_1_	76.4 ± 7.7^&&^	73.1 ± 7.6^&&*^	71.8 ± 6.9^&&**^	0.018
	T_2_	79.4 ± 9.5^&&^	76.5 ± 10.8^&&^	73.2 ± 6.5^&&**^	0.010
	T_3_	84.6 ± 13.4	80.6 ± 13.2	76.9 ± 7.2^&&**^	0.014
	T_4_	82.6 ± 8.8^&&^	83.1 ± 9.5	80.7 ± 6.4^&&^	0.392
HR, beats/min	T_0_	80.0 ± 11.3	77.3 ± 10.0	78.7 ± 10.4	0.511
	T_1_	71.1 ± 7.9^&&^	69.3 ± 7.1^&&^	72.6 ± 14.2	0.345
	T_2_	72.8 ± 10.9^&^	71.8 ± 12.0^&^	75.4 ± 14.0	0.400
	T_3_	75.3 ± 10.0	75.8 ± 14.5	78.2 ± 11.3	0.526
	T_4_	76.7 ± 7.9	78.2 ± 10.8	76.1 ± 9.5	0.601
SpO_2_, %	T_0_	97.8 ± 1.1	97.9 ± 1.1	98.1 ± 1.1	0.476
	T_1_	96.6 ± 2.8^&^	95.5 ± 3.9^&&^	93.8 ± 4.8^&&**^	0.006
	T_2_	96.2 ± 2.4^&&^	95.7 ± 2.9^&&^	95.0 ± 3.8^&&^	0.200
	T_3_	96.9 ± 2.4	96.5 ± 2.3^&&^	96.4 ± 2.2^&&^	0.658
	T_4_	97.8 ± 1.2	98.0 ± 0.9	98.1 ± 0.8	0.482

Compared with group T_0_, ^&^*P* <0.05, ^&&^*P* <0.01; compared with group R2, ^*^P <0.05, ^**^P <0.01

Abbreviations: *HR* heart rate, *MAP* mean arterial pressure, *SpO*_*2*_ pulse oxygen saturation

### HR among groups

**Fig. 5:**
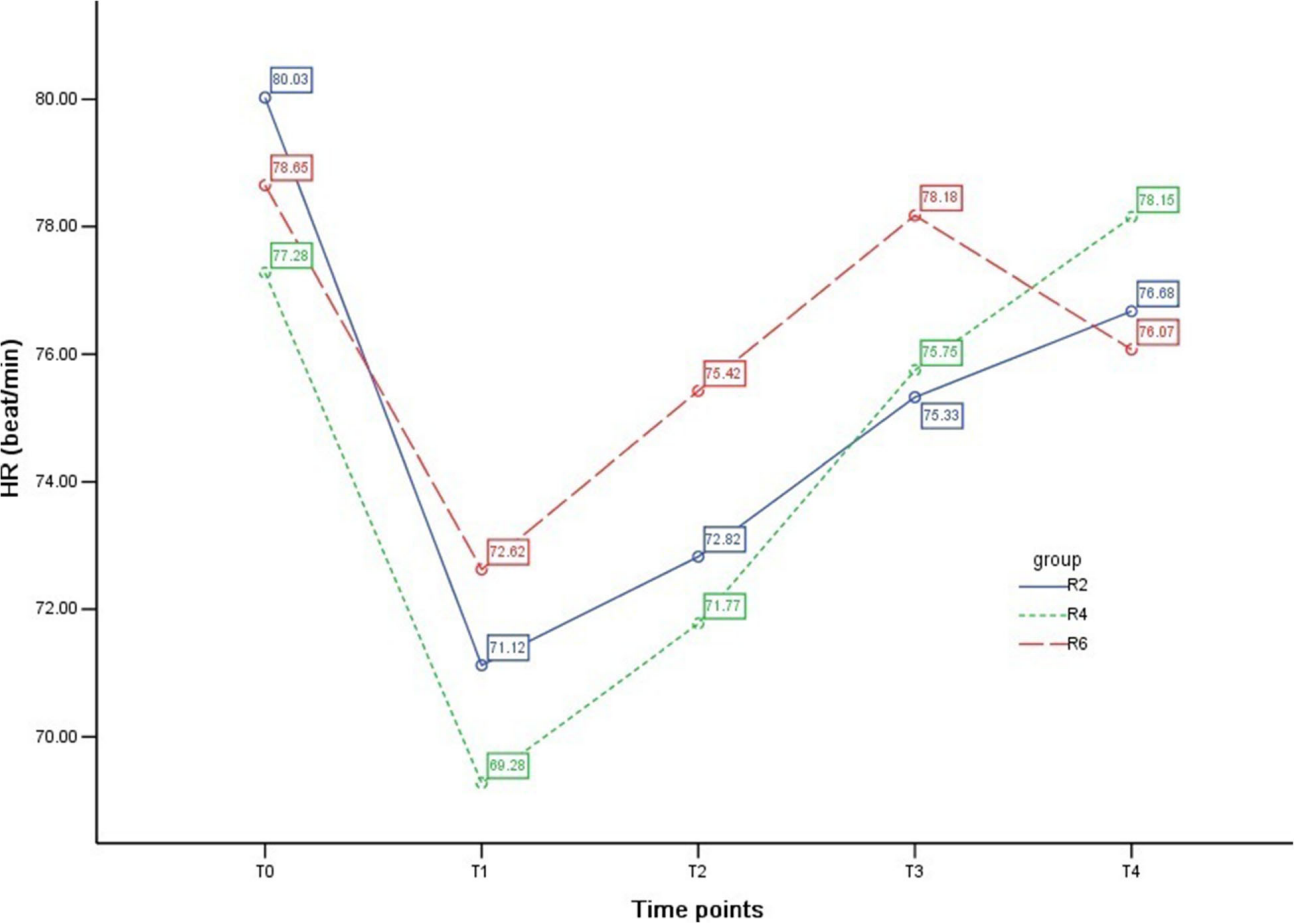
Heart rate (HR) among groups. The variety of HR at different time points in the three groups is shown

HR in the three groups decreased after anesthesia induction, and compared with T_0_, the differences at T_1_ and T_2_ were statistically significant in groups R2 and R4 (*P* <0.05). However, there was no statistical difference among groups (*P* >0.05) (Table 5 and Fig. 5).

### SpO_2_ among groups

**Fig. 6:**
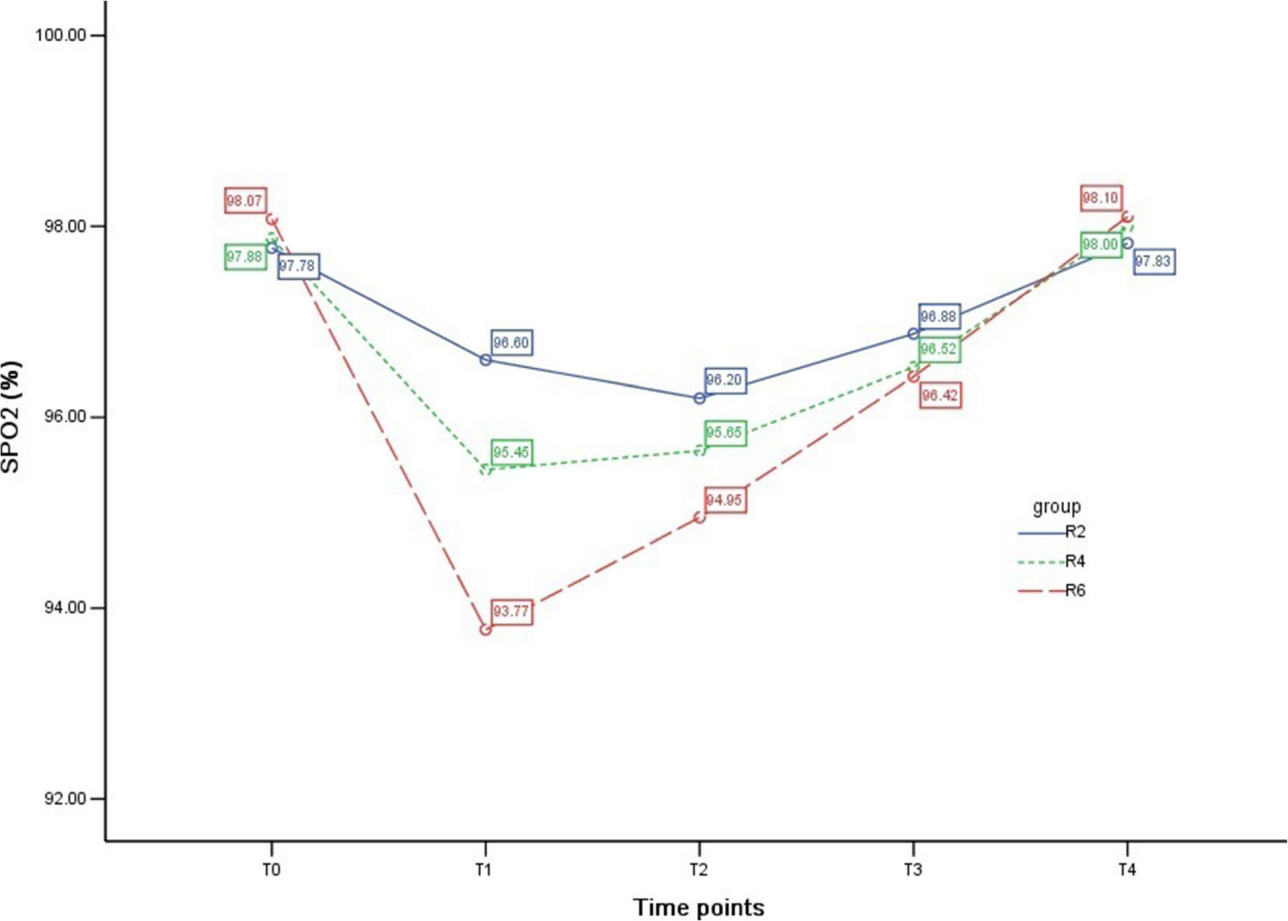
Pulse oxygen saturation (SpO_2_) among groups. The variety of SpO_2_ at different time points in the three groups is shown

Compared with T_0_, SpO_2_ at T_1_ and T_2_ in group R2 and at T_1_, T_2_, and T_3_ in groups R4 and R6 decreased significantly (all *P* <0.01) but came back to the baseline at T_4_. SpO_2_ was relatively lower at T_1_ in group R6 compared with group R2 (*P* <0.01) (Table 5 and Fig. 6).

## Discussion

With the widespread use of target-controlled infusion and anesthesia depth monitoring, accurate anesthesia is gradually becoming possible [34, 35]. The aim of this study was to select a relatively ideal remifentanil target concentration in gastric polypectomy under sedation and analgesia monitoring to help the anesthesiologists lessen the side effects of this agent.

Gastroscopic polypectomy has been performed under general anesthesia for years. The most common anesthetic used in this procedure is propofol [36]; however, there is no analgesic effect of this agent, but the injection pain incidence is as high as about 24% to 86% [37–39], which leads to a reduction in patients’ comfort and cooperation. Remifentanil is a potent narcotic analgesic with rapid onset, high clearance, and short duration [40]. As research progressed, it is reported that the combination of remifentanil and propofol not only can effectively reduce the incidence of propofol-induced pain but also can reduce the dosage of propofol, shorten patients’ recovery time, and enhance patients’ satisfaction in endoscopy [32, 40]. However, the usage of remifentanil varies widely in different studies (for instance, a target concentration of about 0.72 to 3.19 ng/mL [13], a single dose from 0.2 to 0.5 μg/kg [14, 15], and a continuous infusion rate of 6 μg·kg^− 1^·h^− 1^ [33]). As both propofol and remifentanil may cause some dose-dependent respiratory and cardiovascular depression, leading to hypoxemia and hemodynamic instability [40, 41], it is important to have safe and convenient anesthesia depth monitoring to help control anesthetic dose such as remifentanil target concentration during endoscopic therapy.

IOC is generated by the conversion of electroencephalogram (EEG) signals with a symbolic dynamics method, which finally turned into numerical values via an Adaptive Neurofuzzy Inference System that established the relationship between variables on the basis of a non-restricted mathematical structure [42, 43]. As previous research has shown, IOC_1_ can accurately reflect the patient’s sedation state with good consistency with bispectral index [44]. IOC_1_ ranges from 0 to 99; when the value exceeds 80, the patient is generally considered to be awake; about 60 to 80 indicates mild sedation, which reminds the anesthesiologist to add sedative agents; about 40 to 60 represents appropriate sedative depth; and less than 40 indicates immoderate depression [45]. IOC_2_ is derived from IOC_1_, also ranges from 0 to 99, but mainly reflects the degree of analgesia; a value beyond 50 usually signifies inadequate analgesia, 30 to 50 indicates a suitable analgesia state, and below 30 represents excessive analgesic effects [17]. This trial was designed to provide a better and safer analgesia project by comparing the variety of IOC_2_ and the occurrence rate of adverse events with different remifentanil dosages in gastroscopic polypectomy.

Like the constantly changing curves of EEG, the values of IOC_1_ and IOC_2_ fluctuate slightly even at the same concentration of anesthetics during the maintenance of general anesthesia. Given the time delay effect of IOC, it is recommended that the best opportunity to adjust anesthetics was when the values surpass their current level over 20 within 1 min or went beyond their recommended appropriate range for more than 2 min [17, 45]. In this research, each propofol adjustment was guided by the sudden fluctuation of IOC_1_ over 20 within 1 min or remaining above 60 or below 40 for more than 2 min. The results showed that all patients completed the endoscopic treatment successfully, no one had temporary change of treatment, and no patient appeared to have intra-operative awareness, indicating that a plasma concentration of remifentanil of about 2 μg/mL to 6 ng/mL could be used safely in gastric polypectomy. Nevertheless, our study also showed that the incidence of respiratory and cardiovascular depressions increased with the increase of remifentanil dose, indicating that the maximal dosage of remifentanil should be controlled.

In group R2, only one patient had hypoxemia, and the incidence of each single indicator of cardiovascular instability was no more than 20%, suggesting that a dose of remifentanil 2 ng/mL was safe during gastroscopic treatment. However, with the intense stimulation of the therapy, although IOC_1_ remained below 60, this analgesia dosage was relatively insufficient because the average value of IOC_2_ was above 50 and two patients appeared to have body movements during the treatment, indicating that a higher dosage of remifentanil was needed during the period of the resection of gastric polyps under gastroscope.

In group R6, no one experienced hypertension or body movements, signifying a thorough suppression of the adverse nervous reflex. However, with the dose-dependent inhibition on respiratory and cycle systems of remifentanil, the incidence of hypoxemia and bradycardia increased up to 20%, the occurrence of hypotension even reached as high as 30%, and IOC_2_ dropped to below 30 at T_2_, revealing that a concentration of remifentanil 6 ng/mL was excessive. On the other hand, with the potent analgesia and short-acting characteristics of remifentanil, the average dose of propofol decreased and therefore the patient’s awakening time was not delayed.

Compared with patients in groups R2 and R6, those in group R4 showed comparatively stable breathing and hemodynamics, and IOC_1_ and IOC_2_ were maintained at the recommended appropriate ranges. Thus, a plasma concentration of remifentanil 4 ng/mL may be the comparatively ideal dose in gastroscopic polypectomy.

There were some limitations in our study. To begin with, we chose patients undergoing gastroscopic polypectomy because pain intensity was relatively stable throughout the duration of therapy, and the impacts from operation interference among patients were tiny. However, the results were limited and could not be directly extended to other surgeries, and advancement should be made in other surgeries and treatments. In addition, the calculation of the sample size in this study was based on the literature reports related to remifentanil used in gastroscopy. A larger sample and more remifentanil concentration gradients are necessary to help select a more accurate dosage. Moreover, our study found that there was about a 1-min delay in the value reduction of IOC_2_ after anesthesia induction and that the IOC_2_ value might decline with the decrease of IOC_1_, even before the injection of remifentanil, indicating that changes in IOC_2_ were somehow affected by IOC_1_. Therefore, some other anesthesia depth-monitoring parameters (for instance, the bispectral index) should be included in future studies to correlate the parameter with IOC_2_. Finally, the remifentanil concentration in this study was invariable; other methods of anesthetic administration such as “Adjust the dosage of remifentanil according to the changes of IOC_1_ and IOC_2_ during the treatment” should be examined in future studies to help develop a more ideal concentration range of remifentanil.

## Conclusions

In brief, the combination of remifentanil and IOC monitoring is safe, and with the help of IOC monitoring, we found that a target concentration of remifentanil 4 ng/mL is comparatively ideal in patients under gastroscopic polypectomy.

## Abbreviations

ANOVA: Analysis of variance
EEG: Electroencephalogram
HR: Heart rate
IOC: Index of consciousness
IOC_1_: Index of consciousness 1
IOC_2_: Index of consciousness 2
MAP: Mean arterial pressure
NIBP: Non-invasive blood pressure
SpO_2_: Pulse oxygen saturation
